# Effects of Elevated Grids on Growing Male Chickens Differing in Growth Performance

**DOI:** 10.3389/fvets.2019.00203

**Published:** 2019-06-25

**Authors:** Julia Malchow, Birger Puppe, Jutta Berk, Lars Schrader

**Affiliations:** ^1^Institute of Animal Welfare and Animal Husbandry, Friedrich-Loeffler-Institut, Celle, Germany; ^2^Institute of Behavioural Physiology, Leibniz Institute for Farm Animal Biology, Dummerstorf, Germany; ^3^Behavioural Sciences, Faculty of Agricultural and Environmental Sciences, University of Rostock, Rostock, Germany

**Keywords:** broiler, enriched environment, growth performance, platform, growing chickens, walking ability, animal welfare

## Abstract

Pullets, i. e., chickens of layer lines are often raised in housings equipped with perches. In contrast, broiler chickens most often are raised in a barren environment that lacks any three-dimensional structures, even though broilers also are motivated to use elevated structures. In addition, environmental enrichment may improve welfare problems in broiler chickens, such as skeletal disorders or contact dermatitis. Due to ethical reasons, currently there are attempts to fatten the male chickens of layer strains or to use dual purpose strains. However, there is only limited knowledge on the behavior of these chickens until now. The aim of this study was to test the use of elevated grids and their effect on animal-based indicators (e.g., physical condition). In two successive trials, we kept a total of 1,217 male chickens from three strains (Lohmann Dual, Lohmann Brown Plus, Ross 308) that show differences in growth performance in 24 pens (two trials × three strains × eight pens). In half of the pens, grids were offered at three different heights (enriched groups); in the other half of the pens, no elevated structures were installed (control groups). We recorded the number of birds using the grids at the different heights as well as locomotor activity, walking ability, plumage cleanliness, and the footpad health of chickens. Chickens with low and medium growth performance preferred the highest grids during both the light and dark periods. In contrast, fast-growing chickens used the lowest grid more frequently. Fast-growing chickens kept in the enriched pens tended to have a higher level of locomotor activity and reduced chest cleanliness. Chickens from the medium growth performance strain showed better walking ability when kept in the enriched pens. Enrichment did not affect any of the welfare measures in the slow-growing chickens. These findings suggest that elevated structures may improve chicken welfare, particularly for medium growing chickens. For fast-growing chickens we found evidence for an improvement of animal-based indicators although they used the elevated structures less. However, regardless of growth performance, elevated grids offer the birds an opportunity to rest in a species-specific manner.

## Introduction

Broiler chickens commercially are kept in a barren environment, equipped only with littered floors, feeders, and drinkers. Growing chickens are motivated to use and explore elevated structures (1) and such structures can increase their level of activity and improve health of broilers (2). In addition, elevated places may offer chickens shelter in case of fear-eliciting situations (3), as suggested by the antipredator hypothesis (4). Despite these advantages, they are rarely offered in commercial broiler chicken farms.

In previous studies, different elevated structures, such as perches, platforms, straw bales, or bars, have been provided (5–7). It is known that fast-growing broilers prefer platforms compared to perches (8, 9). In studies on height preferences, perches were most often installed at higher levels, whereas grids were installed only at heights up to 30 cm. In a recent study (9), chickens from three strains that differ in growth performance preferred the highest structures, at 50 cm, during the dark period.

In addition to the behavioral restrictions in barren environments, skeletal disorders, and contact dermatitis (10) are common welfare problems in broiler chickens. High growth rate and the associated muscle growth, including that of large breast muscles, lead to cranial shifts in body balance (11) and skeletal disorders (12). Often, fast-growing chickens show impaired walking ability (13), and increased time of rest (e.g., sit/lie) in the litter (10). At the end of the rearing period, the litter is often moist or wet, a result of the high feed intake, metabolism, and excretion of the broilers (14). High resting duration combined with being in contact with moist litter can cause different types of contact dermatitis, including footpad dermatitis, hock burns, and breast blisters (14).

An enriched environment, such as one with elevated structures, can improve the broiler chickens' locomotor activity, and can lead to better walking ability (15). Moreover, broiler chickens that are more mobile and reduce their time resting in contact with litter may show a lowered prevalence of contact dermatitis (16). Furthermore, it has been suggested that offering an elevated structure can result in a cleaner state of the chickens' plumage due to less contact of the keel with the litter (17).

In contrast to fast-growing broiler chickens, chickens of layer strains often are raised with environmental enrichments (18, 19). The main reason is, that pullets shall be prepared for the housing systems in which they will be kept as laying hens, for example in aviary systems with elevated tiers and perches (20). Currently, there are attempts to also fatten the male chickens from layer strains although their weight gain is significantly lower compared the broiler strains. This is done in order to avoid killing of male day-old laying chickens (21) which has increasingly become an ethical issue. A further approach is to use dual-purpose chickens where the female birds are used for egg production and the male chickens for meat production. However, there is still a lack of knowledge on the behavior and in particular on the use of elevated structures by male chickens of both layer and dual strains (22).

In our study, we offered plastic grids at three different heights with a ramp in between to enable easy access. We used three strains to assess possible differences in the use of elevated structures between fast-, medium-, and, slow-growing male chickens. To evaluate the effects of elevated grids, locomotor activity, walking ability, weight, plumage cleanliness, and footpad dermatitis were assessed and compared between unenriched (control groups) and enriched pens.

We predicted there would be a preference for the highest level of the offered grids in all three strains at the end of observation period, at least during dark periods. We expected an improvement in animal-based indicators in the group with access to the elevated grids compared to the control group. In particular, we hypothesized that chickens from enriched pens would show: (a) increased activity; (b) better walking ability; (c) same or higher weight; (d) better scores for total plumage and chest cleanliness, and a worse score for back cleanliness as well as (e) better footpad health.

## Materials and Methods

### Birds and Housing

A total of 1,217 one-day-old male chickens from three different strains were randomly allocated to 12 pens in a stable at the research station of the Institute of Animal Welfare and Animal Husbandry (FLI, Celle, Germany). In two successive trials, 412 Ross 308 (fast-growing, commercial meat strain; hereafter, Ross; first trial included 200 chickens, and second trial included 212 chickens), 400 Lohmann Dual (medium-growing, dual-purpose strain; hereafter, Dual; first trial 200 included chickens, and second trial included 200 chickens), and 405 Lohmann Brown Classic (slow-growing, commercial layer strain; hereafter, LB; first trial included 200 chickens, and second trial included 205 chickens) were used for this study.

All chickens were reared in groups of 50–53 animals (depending on the total number of animals delivered) in experimental pens (floor space: 2 × 3 m; height: 2 m). Chickens of each strain were randomly assigned to four pens (two trials × four groups per strain). The Ross chickens were kept for 5 weeks (body weight at hatch: 44.6 ± 0.4 g; body weight at slaughter date: 2,307.45 ± 306.95 g; mortality: 2.2%), whereas Dual (body weight at hatch: 39.6 ± 2.0 g; body weight at slaughter date: 2,265.0 ± 269.75 g; mortality: 1.5%) and LB (body weight at hatch: 37.7 ± 1.7 g; body weight at slaughter date: 1,372.0 ± 122.75 g; mortality: 1.7%) were kept for 10 weeks (all body weight data: average weight ± standard deviation).

Air temperature and ventilation were automatically controlled with an intermediate program to meet the climate demands of broiler and layer chickens (temperature: 36°C at the first day continuously decreasing to 18°C until 36th day). For the first 3 days, the artificial light program started with a 24 h light period and changed to an 8 h dark period and a 16 h light period (04:00 am to 08:00 pm) at a light intensity of at least 20 lx, including 15 min dimming phases achieved by flicker-free tube-bulbs for the entire experimental periods of both trials.

**Figure 1:**
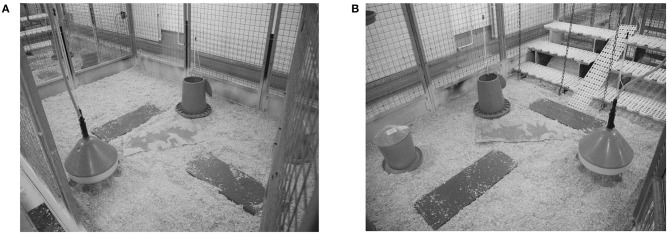
Pens without (**A**, control) and with elevated structures (**B**, enriched). All pens were equipped with wood shavings, two antennas in the litter, two round feeding troughs, and one water dispenser.

Floors of all pens were littered with wood shavings. At the 4th week of age, the litter of Ross chickens was supplemented with chopped straw to keep the litter dry. Two round feeding troughs and one round water dispenser with eight drinking nipples were provided per pen (Figures 1A,B). All chickens were fed *ad libitum* with a single-phase pelletized feed (21% crude protein, 12.90 MJ ME/kg) that met the nutritional needs of both broiler and layer chickens.

In both trials, half of the pens of each strain were equipped with elevated grids at three different heights (10, 30, 50 cm) but with the same shape (length × width: 90 × 30 cm). A ramp (width: 20 cm, inclination angle: 35°) was installed in between the grids to provide easy access (Figure 1B). Both the grids and ramps were made of the same material [plastic: PP (Polypropylene)] and had a mesh size of 19 × 19 mm and a slat width of 10 mm.

### Measurements and Statistical Analysis

All statistical analyses were done using SAS^®^ Enterprise Guide Version 6.1. To test the effects of factors and their interactions, we used adapted generalized linear mixed models (GLMM). For examining significant effects, we used *post hoc* tests (Bonferroni) for testing pairwise differences based on our hypotheses. In each calculation, pen ID was included as a random factor nested within the random factor trial.

### Use of Structures

In the enriched pens, the elevated structures were recorded using infrared video cameras (Model VTC-E220IRP, color camera for corner mount with IR-LEDs; SANTEC BW AG, Ahrensburg, Germany) connected to a commercial PC with memory function. From these recordings, the numbers of chickens on the grids were counted for each height using time sampling in 20-min intervals for each week of age throughout two successive days (usually Saturday and Sunday) from 03:00 a.m. to 10:00 p.m.

To test preferences for the height of elevated structures (10, 30, 50 cm) in the enriched pens (*n* = 12), each observation day was divided into a light period (from 04:00 a.m. to 07:40 p.m.) and a dark period (from 03:00 a.m. to 03:40 a.m. and from 08:00 p.m. to 10:00 p.m.). As the dependent variable, we calculated the mean proportion of chickens at each height, week, and strain separately for light and dark periods, excluding the number of chickens on the ramp. Height, week of age, strain, period, and their interactions were used as fixed factors.

### Locomotor Activity

The locomotor activity was automatically recorded with a transponder-antenna system (PLB SPEED Antenna and Chip Glastag HITAGS 3.15 × 13 × 3 mm; Gantner Pigeon Systems GmbH, Schruns, Austria). In each pen, two antennas (length: 90 cm, width: 30 cm, height: 3 cm) were placed on the floor in the litter (Figures 1A,B). At an age of 14 days, half of the chickens in each pen had a transponder attached (height: 23 mm, width: 4 mm, weight: 2.5 g) with a cable strap to their legs. In addition, the chickens received a chicken tag with an individual number at the wing and, thus, in case a chicken lost the transponder, a new one could be assigned.

The antennas recorded the transponders at a distance of <20 cm and a connected computer registered the number of the antenna and the transponder tag, as well as the date and time. As a proxy for the locomotor activity for each transponder (chicken), we calculated the mean number of changes between the two antennas per week for each chicken transponder using SAS^®^ 9.4 (SAS Inst. Inc., Cary, NC) during the light period (04:00 a.m. to 08:00 p.m.).

For testing the effects of treatment (enriched/ control) on the locomotor activity measured by the mean frequency of changes between antennas, we used treatment, strain and week of age as fixed factors.

### Walking Ability

Walking ability was assessed with the rotarod test 2 days before slaughter. The data of the rotarod test covary with the results obtained by the gait score system (23) and, thus, offer a more objective assessment of walking ability. In short, a chicken was placed in the middle of a rod. After both feet grasped around the rod, the motor that rotates the rod was started. The test stopped when the chicken actively or passively left the rotating rod. The details of this test are described in Malchow et al. (24).

Chickens with a transponder were chosen in a random order from each pen. For each strain, we tested 46 birds from both trials (six chickens from three pens, and five chickens from one pen). The number of animals based on preliminary tests.

To test the effect of treatment (enriched/ control) on walking ability, we measured the latency to leave the rotating rod. Treatment, strain and their interaction were used as fixed factors.

### Weighing and Assessment of Plumage Cleanliness and Footpad Health

All chickens that were equipped with a transponder were weighed (nearest ± 10 g), and their plumage cleanliness and level of footpad health were assessed at the end of the rearing period.

Plumage cleanliness and footpad health were assessed with the Welfare Quality protocol for poultry (25). The scoring system for the plumage was classified in four categories: 0—no contamination; 1—light contamination; 2—moderate contamination; and 3—high contamination with litter glued to feathers. The categories were assessed on five different parts of the chickens: head/neck, back, tail, wings, and chest. To evaluate footpad health, we used a five-scale system: 0—no changes (no evidence of footpad dermatitis); 1—light changes of the footpad (slightly evidence of footpad dermatitis); 2—moderate changes of the footpad (minimal evidence of footpad dermatitis); 3—entire footpad shows changes; and 4—changes of the entire central footpad and also of the plantar toes (evidence of footpad dermatitis) (25).

For testing the differences between the weights of chickens from enriched and control groups, we used strain, treatment and their interaction as fixed factors.

The total plumage cleanliness scores for three (head, tails, wings) body parts for each pen were added up to a total score ranging from 0 to 9. The lower the total score, the better the plumage cleanliness was. Effects on the total score for cleanliness were tested by including strain, treatment, and their interaction as fixed factors. With respect to plumage cleanliness, we expected differences between cleanliness particularly for the back and the chest plumage. Thus, for these two body parts and footpad health, we did a separate analysis using a Mann Whitney *U*-test as data were not normally distributed and statistics were done for each strain.

## Results

### Use of Structures

**Figure 2:**
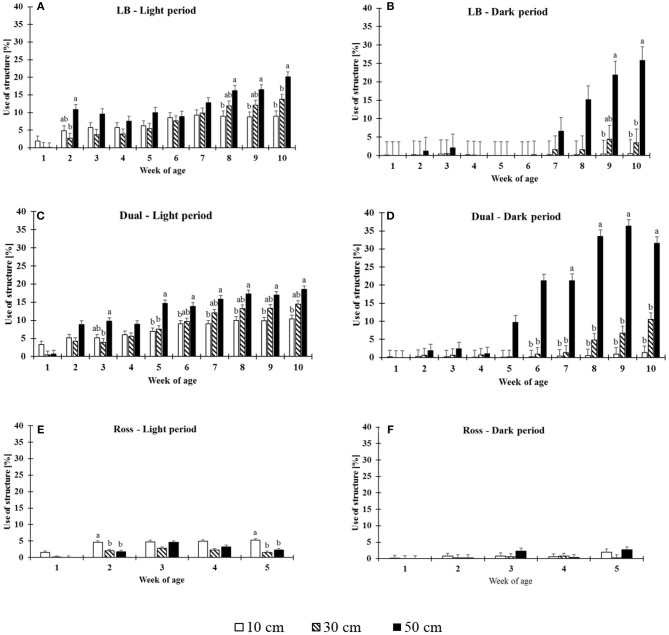
Proportion of the use of structures of different heights (LSM ± SEM) by chickens from three strains [LB **(A,B)**, Dual **(B,C)**, Ross **(D,E)**] during the light **(A,C,E)** and dark periods **(B,D,F)**. Significant differences between heights within week of age are marked by different letters (*P* < 0.05).

The use of structures was significantly affected by 2-fold interactions between strain and period (*F*_(13, 528)_ = 8.92, *P* = 0.0002), strain and height (*F*_(4, 528)_ = 7.16, *P* < 0.0001), week and period (*F*_(9, 528)_ = 2.7, *P* = 0.0044), week and height (*F*_(18, 528)_ = 10.63, *P* < 0.0001), and period and height (*F*_(2, 528)_ = 23.46, *P* < 0.0001). LB and Dual chickens used the elevated platforms more with increasing age both during the daytime and at night (Dual: dark period: *F*_(9, 87)_ = 33.02, *P* ≤ 0.0001, light period: *F*_(9, 87)_ = 58.12, *P* < 0.0001; LB: dark period: *F*_(9, 87)_ = 3.47, *P* = 0.0011, light period: *F*_(9, 87)_ = 38.80, *P* < 0.0001). Ross chickens showed a very low use compared with the chickens from the slower growing strains and usage of structure was only affected by the week of age during light period in Ross chickens (dark period: *F*_(4, 42)_ = 1.94, *P* = 0.122; light period: *F*_(4, 42)_ = 35.54, *P* < 0.0001, Figure 2).

The maximum proportion of animals (LSM ± SE) on the elevated grids varied from 5 ± 0.44% (Ross) in the light period to 26 ± 3.75% (LB) and to 36 ± 1.83% (Dual) except in Ross during the dark period. The height significantly affected the use of the grids (except in Ross at dark period, see Table 1). In general, Dual and LB chickens primarily preferred the highest grids both during the light and dark periods in the middle and at the end of the observation period (Figure 2). In contrast, in the light period, Ross chickens preferred the lowest level of elevated platforms. During the dark period, Ross birds showed no preference for any of the three heights.

**Table 1:** Interaction between the heights of the structure and the week of age on the frequency of structure usage within strains and daytime (GLMM, factor height*week of age).

**Strain**	**Daytime**	**numDF**	**denDF**	***F*-statistic**	***p*-value**
LB	Light period	18	87	3.47	<0.0001
	Dark period	18	87	2.18	0.0087
Dual	Light period	18	87	3.49	<0.0001
	Dark period	18	87	18.50	<0.0001
Ross	Light period	8	42	3.00	0.0094
	Dark period	8	42	0.80	0.6031

### Locomotor Activity

**Figure 3:**
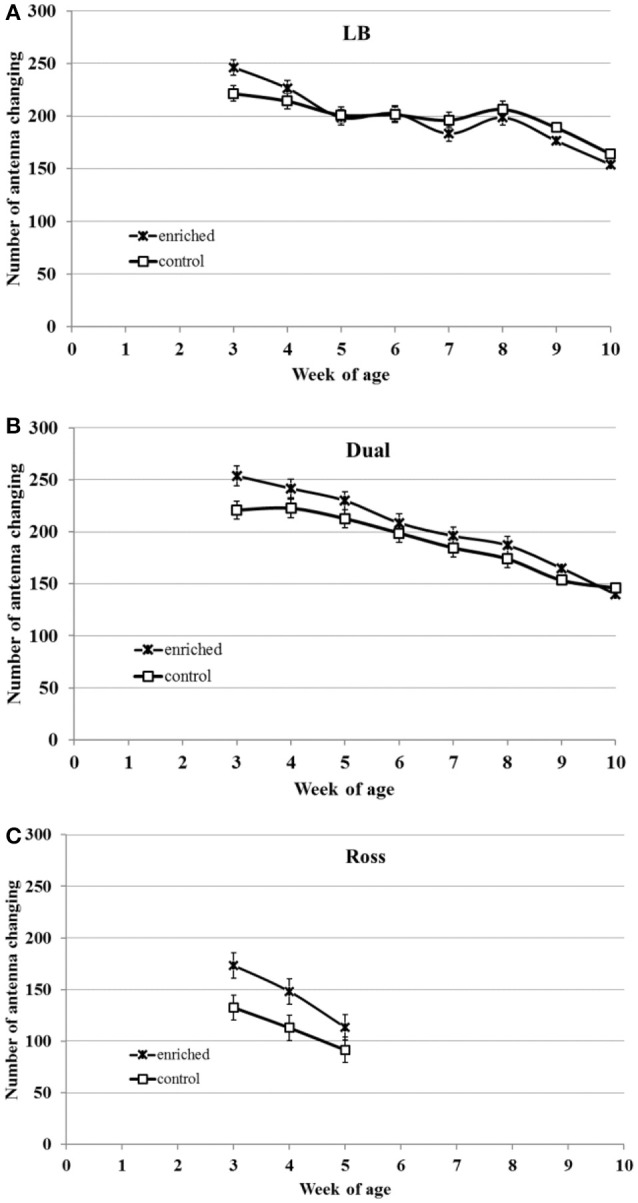
Locomotor activity (LSM ± SEM) of chickens from three strains [**(A)** LB, **(B)** Dual, and **(C)** Ross] with (enriched) and without (control) elevated structures. The locomotor activity was measured in means of the number of changes between two floor antennas.

Regardless of environmental enrichment, all three strains showed decreasing activity with increasing age (LB: *F*_(7, 42)_ = 23.81, *P* < 0.0001; Dual: *F*_(7, 41)_ = 40.72, *P* < 0.0001; Ross: *F*_(2, 12)_ = 67.44, *P* < 0.0001, Figure 3). There was a significant interaction between strain and week of age (*F*_(9, 95)_ = 3.13, *P* < 0.0001), and between treatment and week of age (*F*_(9, 95)_ = 3.08, *P* = 0.0057).

In LB chickens, the treatment showed no effect on activity. Dual (*F*_(1, 41)_ = 3.42, *P* = 0.072) and Ross chickens (F_(__1, 12)_ = 3.87, *P* = 0.073) tended to show higher activities in the enriched group compared to the respective control group.

### Walking Ability

**Figure 4:**
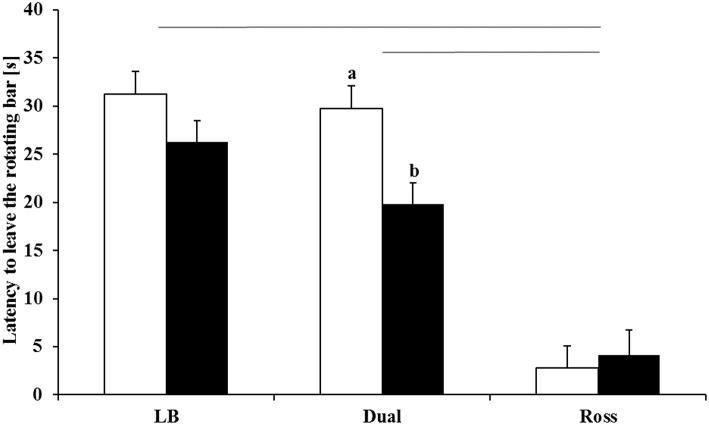
Latency to leave the rotating bar (LSM ± SE) in the rotarod test for LB, Dual, and Ross chickens housed with (enriched, white bars) and without (control, black bars) elevated structures. Significant differences between enriched and control pens are marked by different letters (*P* < 0.05). Significant differences between strains and treatments are marked by lines (*P* < 0.05).

Walking ability was significantly affected by the interaction between treatment and strain (*F*_(2, 114)_ = 3.12, *P* = 0.0478). LB and Dual chickens showed a comparable latency to leave the rotating rod (*P* = 0.2397). Ross chickens showed a worse walking ability than Dual (*P* < 0.0001) and LB (*P* < 0.0001) chickens. In Dual chickens, birds from the enriched groups showed a significantly longer latency to leave the rotating rod than chickens from the control groups (*P* = 0.0346, Figure 4). Ross and LB had no differences between the treatments.

### Weight, Plumage Cleanliness, and Footpad Health

The treatment did not affect the weight of chickens (*F*_(1, 18)_ = 0.14, *P* = 0.87).

**Figure 5:**
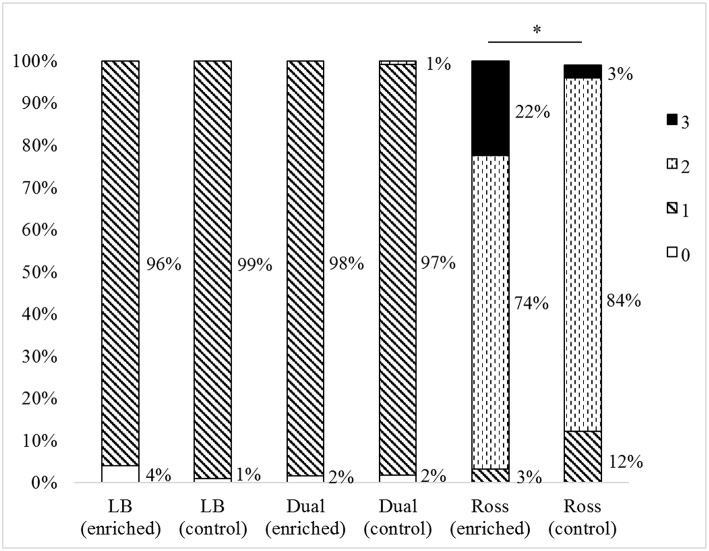
Chest cleanliness scores (percentage of animals assessed for each score) of the three strains (LB, Dual, Ross) for the two treatments (enriched and control pens). Significant differences between treatments within strains are marked by an asterisk, *P* < 0.05.

We found differences in the total cleanliness score of the plumage between strains (*F*_(2, 597)_ = 24.33, *P* < 0.0001), but no differences between the treatments (total cleanliness score: *F*_(1, 597)_ = 0.15, *P* = 0.7). In general, Ross chickens were dirtier than Dual (*P* < 0.0001) and LB (*P* < 0.0001), and Dual more than LB (*P* = 0.0414). Only Ross chickens showed differences between the treatments in back and chest cleanliness. Both body parts were dirtier in the enriched groups than in control groups (back: *Z* = 36.43, *P* = 0.0563; chest: *Z* = 200.39, *P* < 0.0001, Figure 5).

Footpad health was only affected by treatment in Dual chickens (*Z* = 6.1019, *P* = 0.0135). Dual chickens from the control groups showed worse footpad health compared to chickens from the enriched groups. Footpad health significantly differed between strains in the control groups (Ross > Dual > LB, *Z* = 143.87, *P* < 0.001) and in the enriched groups (Ross > Dual = LB, *Z* = 149.44, *P* < 0.0001). In sum, Ross showed worse footpad health in comparison to Dual and LB.

## Discussion

In general, chickens of all three strains showed increasing use of elevated structures with increasing age during both the light and dark periods. This result confirms the outcomes of other studies conducted with fast-growing chickens (6, 8). In general, growing chickens' use of elevated structures may result from the motivation to rest, sleep and explore on high levels (1). Another explanation could be that they may use the structures to avoid agonistic interactions with dominant conspecifics, as observed in laying hens (26).

In our study, chickens with slower growth (LB and Dual) additionally showed mostly a preference for the highest grids both during the day and night time. This preference corresponds to the preference for layers for high perches (9, 27–29) and can be explained by the antipredator hypothesis (4) suggesting that chickens experience better protection from predators if they stay on elevated structures. Although a preference for high resting areas is particularly pronounced in adult fowl for night roosting (27, 28, 30), already growing chickens show a high motivation to stay at high levels overnight (9, 31), which indicates that elevated areas may offer shelter for growing chickens, as well. In contrast to the slow-growing chickens, most of the fast-growing chickens were observed on the lowest level during the light period. This result correlates with the outcomes of the studies of Estevez et al. (32) and of Norring et al. (8) on the use of perches offered to fast-growing broilers at different heights. This outcome likely results from the rapid growth and the resulting reduced locomotor activity of these chickens. At the end of the rearing period, their balance is impaired, which is caused by the high mass of breast muscle that leads to a cranial shift of the body center (11). To facilitate access to the elevated grids, we included a ramp that was most often used in particular by the fast-growing chickens (personal observation). However, the design of the ramp used in our study seemed to be less suitable for use by the heavy broilers. The ramp might have been too steep, the ramp might have been unsteady, or the width of the ramp might have been too small for the fast-growing chickens (only one bird could use the ramp at a time). Climbing up a ramp requires a particular force from the chickens because they need a higher force to take a step and to balance on one foot while climbing against the ascent of the ramp (33). In a previous study (9) we used the same type of ramp and the chickens, especially the fast-growing chickens (Ross), showed higher usage (14 vs. 4%) during both the light and dark periods. However, the chickens of the first study were lighter (2,099 ± 583 g) than the chickens of the present study (2,307 ± 307 g). This heavier weight might have reduced the chickens' ability to balance and climb up the ramp in the present study.

Chickens of all three strains showed decreasing locomotor activity with increasing age. Furthermore, the fast-growing chickens reflected a lower locomotor activity than the medium- and slow-growing chickens. Similar to previous studies (15, 34), our findings suggest that in chickens with a faster growth, environmental enrichment, i.e., elevated structures, had an effect on chickens' locomotor activity. Compared to the slower-growing LB chickens, the fast-growing Ross chickens and the medium-growing chicken Dual tended to have a higher activity in week 3 of observation in the enriched pens compared to chickens in the control pens. Ventura et al. (35) observed a higher activity level when broilers had to cross perches as a barrier in their pens. This larger effect of enrichment in the fast-growing Ross chickens is particularly interesting because they used the elevated grids to a lower degree compared to the slower growing chickens. Thus, this result suggests that in chickens with faster growth, elevated grids seem to increase their activity, even if this type of enrichment is used infrequently. However, although activity is closely associated with walking ability in chickens (36), the fast-growing chickens from enriched pens did not differ from those in control pens in their walking ability, as indicated by the results of the rotarod test. In this test, only Dual chickens were affected by the enrichment, i.e., Dual chickens from enriched pens showed a longer latency to leave the rotating rod compared to the Dual chickens from the control pens. Thus, the enrichment may have only trained the motor abilities of the medium-growing, but not that of the slow- or fast-growing chickens. For fast-growing chickens, this result corresponds to the low use of the elevated structures and their low activity level in both the enriched and control groups. Other studies with fast-growing chickens found either positive (7, 37) or no (12, 38) effects of enrichment on walking ability. The slow-growing chickens (LB) showed a good walking ability with long latencies to leave the rotating bar but did not differ between enrichment treatment. These chickens are from a layer line that shows more mobile and active phenotypes in general. Hence, in LB, the elevated structures used in our study may not further improve their already well-developed motor skills. In comparison, the dual-purpose breed (Dual) used in this study is a crossbreed from a slow- and a fast-growing chicken line (39). Therefore, enrichment with elevated grids seems to have an effect on walking ability only in the medium type of growth performance.

In our study, we did not find differences in weight between chickens from enriched and control groups, which confirmed the results of Bizeray et al. (34) and Simsek et al. (40). Thus, enrichment by elevated structures had no detrimental effect on production efficiency.

We also did not find an effect of elevated structures on the total plumage cleanliness or on the cleanliness of the back. In contrast, the fast-growing chickens showed poorer (higher score) cleanliness of the chest in the enriched groups compared to the control groups. A possible explanation may be that Ross chickens in enriched pens did not use the area under the elevated structures (personal observation), and at the same time, they used the elevated grids a small amount. This outcome may have resulted in a higher density of chickens in the litter (in front of the elevated structures) compared to the control pens in which the respective area was freely accessible for the chickens. Thus, the feces were concentrated within a smaller area of the enriched pens of the fast-growing chickens, which may have resulted in their poorer chest cleanliness scores.

According to our expectation, the elevated grids affected footpad health, but only in Dual chickens. We had expected that by using the grids, the feet of chickens may be healthier because footpad lesions can result from the contact of footpads with moist litter (10). However, footpad health in our study was quite good compared with footpad health observed in commercial housings [prevalence of 42%, Sanotra et al. (41)]. This may explain that we did not find differences in the layer (LB) and the meat (Ross) strain in our study.

In conclusion, chickens from all three strains differing in growth rate used the elevated grids, although strains differed in the usage frequency. In particular, at night, the slower growing strains LB and Dual preferred the highest grids, and even young chickens were motivated to rest and roost on elevated structures. The usage of the elevated grids should to be adapted to the respective strain, as indicated by the results for the fast-growing Ross chickens. The elevated grids used in our study did not have a negative effect on the growth performance of chickens. In contrast, some of the animal-based indicators were improved by the elevated grids, such as locomotor activity and walking ability. However, these positive effects on the chickens' welfare depended on the strain, i.e., the effects interacted with the growth rate of the chickens. Thus, elevated grids seem to better fulfill the behavioral demands of growing chickens but have to be adapted to their skills and abilities, particularly for fast-growing chickens, in order to improve their welfare.

## Data Availability

All datasets generated for this study are included in the manuscript and/or the supplementary files.

## Ethics Statement

This study was performed in compliance with national regulations (TierSchNutztV as of 2006) at the research station of the Institute of Animal Welfare and Animal Husbandry (FLI, Celle, Germany). All investigations were carried out with the approval of the Lower Saxony State Of-fice for Consumer Protection and Food Safety (LAVES, Oldenburg, Germany, file number 33.19-42502-04-16/2108).

## Author Contributions

JM, JB, and LS conceived and designed the project. JM collected and analyzed the data. All authors contributed to the manuscript.

### Conflict of Interest Statement

The authors declare that the research was conducted in the absence of any commercial or financial relationships that could be construed as a potential conflict of interest.
